# An integrated perspective linking physiological and psychological consequences of mild traumatic brain injury

**DOI:** 10.1007/s00415-019-09335-8

**Published:** 2019-04-27

**Authors:** Harm Jan van der Horn, Manon L. Out, Myrthe E. de Koning, Andrew R. Mayer, Jacoba M. Spikman, Iris E. Sommer, Joukje van der Naalt

**Affiliations:** 1 Department of Neurology, University of Groningen, University Medical Center Groningen, Hanzeplein 1, 9700 RB Groningen, The Netherlands; 2 Department of Neurology, Medisch Spectrum Twente, Koningsplein 1, 7512 KZ Enschede, The Netherlands; 3 The Mind Research Network, 1101 Yale Blvd NE, Albuquerque, NM 87106 USA; 4 Department of Neuropsychology, University of Groningen, University Medical Center Groningen, Hanzeplein 1, 9700 RB Groningen, The Netherlands; 5 Department of Neuroscience, University of Groningen, University Medical Center Groningen, Antonius Deusinglaan 2, 9713 AW Groningen, The Netherlands

**Keywords:** Mild traumatic brain injury, Biomarkers, Cytokines, Cortisol, Psychology

## Abstract

Despite the often seemingly innocuous nature of a mild traumatic brain injury (mTBI), its consequences can be devastating, comprising debilitating symptoms that interfere with daily functioning. Currently, it is still difficult to pinpoint the exact cause of adverse outcome after mTBI. In fact, extensive research suggests that the underlying etiology is multifactorial. In the acute and early sub-acute stages, the pathophysiology of mTBI is likely to be dominated by complex physiological alterations including cellular injury, inflammation, and the acute stress response, which could lead to neural network dysfunction. In this stage, patients often report symptoms such as fatigue, headache, unstable mood and poor concentration. When time passes, psychological processes, such as coping styles, personality and emotion regulation, become increasingly influential. Disadvantageous, maladaptive, psychological mechanisms likely result in chronic stress which facilitates the development of long-lasting symptoms, possibly via persistent neural network dysfunction. So far, a systemic understanding of the coupling between these physiological and psychological factors that in concert define outcome after mTBI is lacking. The purpose of this narrative review article is to address how psychophysiological interactions may lead to poor outcome after mTBI. In addition, a framework is presented that may serve as a template for future studies on this subject.

## Introduction

The vast majority (85–90%) of patients with traumatic brain injury (TBI) sustain a mild traumatic brain injury (mTBI). With an estimated annual worldwide incidence of approximately 600/100,000 persons (42,000,000 persons worldwide), mTBI is the most common neurologic disorder [1, 2]. For a considerable percentage of patients (≈ 25%) it takes months or even years to recover due to persistent cognitive and emotional symptoms that interfere with resumption of work, social activities and studies [3–5]. Hence, this patient group imposes a tremendous burden on health services and economy. Although acute symptoms are thought to result from the brain injury itself, a comprehensive physiological substrate for the persistence of symptoms has not been empirically established. In fact, for the majority of patients conventional computed tomography (CT) or magnetic resonance imaging (MRI) do not show traumatic abnormalities, and if abnormalities are identified, they tend to correlate poorly with persisting symptom severity [6–8].

The application of advanced imaging modalities, such as functional MRI (fMRI) and diffusion-weighted imaging (DWI), has provided evidence of altered brain network connectivity after mTBI, although a clear-cut mechanism underlying continued symptoms has not been found [9, 10]. There is also increasing evidence that certain protein biomarkers of cellular injury in the acute phase post-injury are informative of injury severity and clinical outcome after mTBI [11, 12]. However, it is unclear if these markers have a role in the pathophysiology of persistent symptoms, and how this process may be influenced by other acute physiological sequelae such as inflammation or the acute stress response. Furthermore, there still exists a lively debate as to whether the influence of psychological factors may outweigh that of the physiological consequences of the injury itself in the causation of persistent symptoms and poor outcome after mTBI. Psychological factors, such as the presence of anxiety and depression, and the employment of certain coping styles, are known to exert a strong influence on recovery after mTBI [5, 13–15]. Not surprisingly, stressful events such as sustaining a traumatic brain injury, can result in an increased demand on coping skills, especially in the current busy and often stressful modern environment. One of the most important elements of coping is the ability to regulate negative emotions and stress, which is closely related to our personality [16, 17]. Inadequate emotion regulation could lead to emotional distress, such as anxiety and depression, which may enhance the persistence of post-traumatic symptoms.

The pathophysiology of mTBI encompasses various (mostly acute) physiological and (mostly sub-acute/chronic) psychological processes, which are intricately linked. Disentangling these relationships forms one of the major challenges in mTBI research. For example, it is still poorly understood whether or not physiological disturbances in the acute phase post-injury, such as cellular injury, inflammation and the acute stress response, are related to psychological problems at a later stage after mTBI, and whether there is an association with perturbations in neural networks that are necessary for emotion regulation and adequate coping skills. In the current narrative review, we aim to provide an overview of these physiological and psychological factors, and theoretically explore the interaction between these factors in the etiology of persistent symptoms and poor outcome after mTBI.

## Cellular injury

Traumatic injury to neuronal, axonal, glial, and vascular structures causes the release of brain-specific proteins [11]. Promising results have been published suggesting that serum levels of brain-specific proteins, such as S100 calcium-binding protein B (S100B; primarily found in astroglial cells, but also in melanocytes), glial fibrillary acidic protein (GFAP; present in the cytoskeleton of several cells in the central nervous system, for example astrocytes), tau (protein that stabilizes microtubuli that make up the cytoskeleton of axons), neurofilament light (NF-L; protein that is also part of the cytoskeleton of neurons), and ubiquitin C-terminal hydrolase-L1 (UCH-L1; an enzyme which plays a role in the repair of neurons and axons via regulation of protein degradation), are predictive of the presence of lesions after mTBI, and poor outcome after mTBI [11, 18–23]. In Scandinavia, S100B measurement in the acute phase post-injury has already been added to the Neurotrauma guidelines as a biomarker to reduce unnecessary CT-scans and associated costs [24]. In the United States, UCH-L1 and GFAP have been approved for the same purpose [23]. In sports-related mTBI, elevated tau-protein is related to recovery time and return to play [18]. It is thought that tau measurements might be used as a tool to detect residual neural damage, even in the absence of acute symptoms, which may protect a player from premature return to play, thereby reducing the risk of sustaining additional injury. Furthermore, hyperphosphorylated tau protein is a hallmark of the pathophysiology of chronic traumatic encephalopathy (CTE), which is a neurodegenerative disorder that can occur even in patients with (repetitive) mild TBI [25].

Despite these findings, we still know little about the exact role of these proteins in the development of persistent symptoms. The kinetics of these biomarkers vary, with S100B and GFAP reaching a peak serum concentration in the first 24 h after injury (‘acute biomarkers’), tau staying elevated for days to weeks (‘subacute biomarkers’), and NF-L continue rising for weeks to months after injury (‘chronic biomarkers’) [26]. Therefore, it would be interesting to investigate whether ‘acute’ biomarkers predict, and if ‘chronic’ biomarkers accompany long-lasting symptoms.

## Inflammation

Under normal circumstances, inflammation is closely regulated, and helps to repair damaged tissue and to fight infections [27, 28]. However, excessive and prolonged inflammation, for example after TBI, has the opposite effect [28]. It has been shown that neuroinflammation occurs within seconds to minutes after a TBI, and involves a complex cascade of microglia activation, pro- and anti-inflammatory cytokine release, and up- and downregulation of neurotrophic factors [28, 29]. Neuropathological research has demonstrated signs of neuroinflammation up to 18 years post-injury in patients with moderate/severe TBI, and these findings were related to white-matter degeneration [30]. Furthermore, a positron emission tomography (PET) study on American football players has shown microglia activation many years after the last mTBI [31], and chronic microglia activation is associated with the development of CTE [32]. Studies using rodent models have yielded data on the relationship between inflammation and behavioural deficits after mild TBI [29, 33–37]. However, relatively little is known about the role of inflammation in the pathophysiology of sequelae following mild TBI in humans, as compared to moderate-severe TBI [29]. A retrospective study on a human mTBI sample has shown an association between persistent post-traumatic symptoms and elevated C-reactive protein within the first 72 h after injury [38]. Another recent retrospective study demonstrated a relation between interleukin (IL)-1β and IL-6 gene polymorphisms and the number and duration of post-traumatic symptoms after sports-related concussion [39]. In addition, reviews have been published that posit the possible influence of earlier (pre-injury) pro-inflammatory states, extracranial injury, infections and immunosenescence (i.e., deterioration of the immune system due to aging) on the occurrence of (neuro)inflammation in the pathophysiology of mTBI [40, 41]. For example, similarities exist between post-traumatic symptomatology and cognitive or behavioral changes that arise from other inflammatory conditions, such as sepsis [40]. Interestingly, a recent study has shown that using an unsupervised multivariate approach applied to a multi-analyte proteomic panel, including several inflammatory markers, it was possible to fairly accurately predict positive CT-findings and outcome in a study sample that consisted predominantly of mild TBI patients, although 42% of these patients had lesions on CT [42].

It is important for future studies on the role of acute and chronic inflammation in the development of persistent symptoms and poor outcome, to compare patients with mild TBI to patients with peripheral inflammatory conditions, such as orthopedic injury.

## Stress

During an acute stress response, for example elicited by a traumatic brain injury, brain structures such as the hypothalamus, amygdala, and insula become activated, which engage the sympathetic autonomic nervous system and the hypothalamic–pituitary–adrenal (HPA) axis, leading to increased heart action and the release of catecholamines and cortisol. The release of cortisol is needed to initiate adaptive metabolic and mental processes to deal with the acute stressor [43]. However, an exaggerated and protracted stress response, which may be due to poor emotion regulation skills in case of stressful situations, is harmful to an individual. Chronically changed cortisol levels are associated with various physical and mental diseases [44]. Furthermore, excessive or prolonged stress can induce inflammation, via microglia activation, and the release of pro-inflammatory cytokines such as interleukin (IL)-1β and 6, and tumor necrosis factor (TNF)-α [27, 45]. These cytokines have also been found to be related to emotion regulation deficits in healthy young adults [46]. An important causative factor in the pro-inflammatory effect of (especially chronic) stress is glucocorticoid resistance, which is the decrease in sensitivity of immune cells to the effects of glucocorticoids [47].

There have been some data published on cortisol in patients with mTBI, and patients with mild to severe TBI [48–52]. These studies, although limited by sample size and lack of control groups, demonstrated that associations are present between cortisol levels, injury severity, and cognitive deficits at various stages post-injury. On the one hand, alterations in cortisol levels after TBI can be caused by HPA-axis dysfunction due to the injury itself, which is common in patients with TBI, even following mild TBI [53, 54]. On the other hand, alterations in cortisol levels (i.e., increased levels) after mTBI can be viewed as a physiological response to the psychological effects of sustaining a TBI, which is a stressful event. It is still unknown if acute changes in cortisol levels are involved in the causation and/or persistence of symptoms, and if the effects of stress are mediated via inflammation. In contrast to acute stress, it is thought that psychological mechanisms are responsible for chronic stress and persistent symptoms after mTBI, although to our knowledge this has not yet been corroborated by results from longitudinal cortisol measurements. Similar to cytokine analyses, acute changes in cortisol can be reliably measured in both serum and saliva. Interestingly, chronic stress can (also) be measured in hair samples. In fact, differences in long-term hair cortisol levels have been found between various mental illness such as major depressive disorder, anxiety disorders and post-traumatic stress disorder, illnesses which are also quite common after mild TBI [44, 55]. Since hair grows approximately 1 cm per month, hair analysis is also informative of the cortisol levels before and after a stressful event, which makes it possible to investigate stress before and after mTBI, and more specifically, to investigate whether pre-injury stress levels are associated with chronic stress after injury.

## Neural networks and emotion regulation

**Fig. 1:**
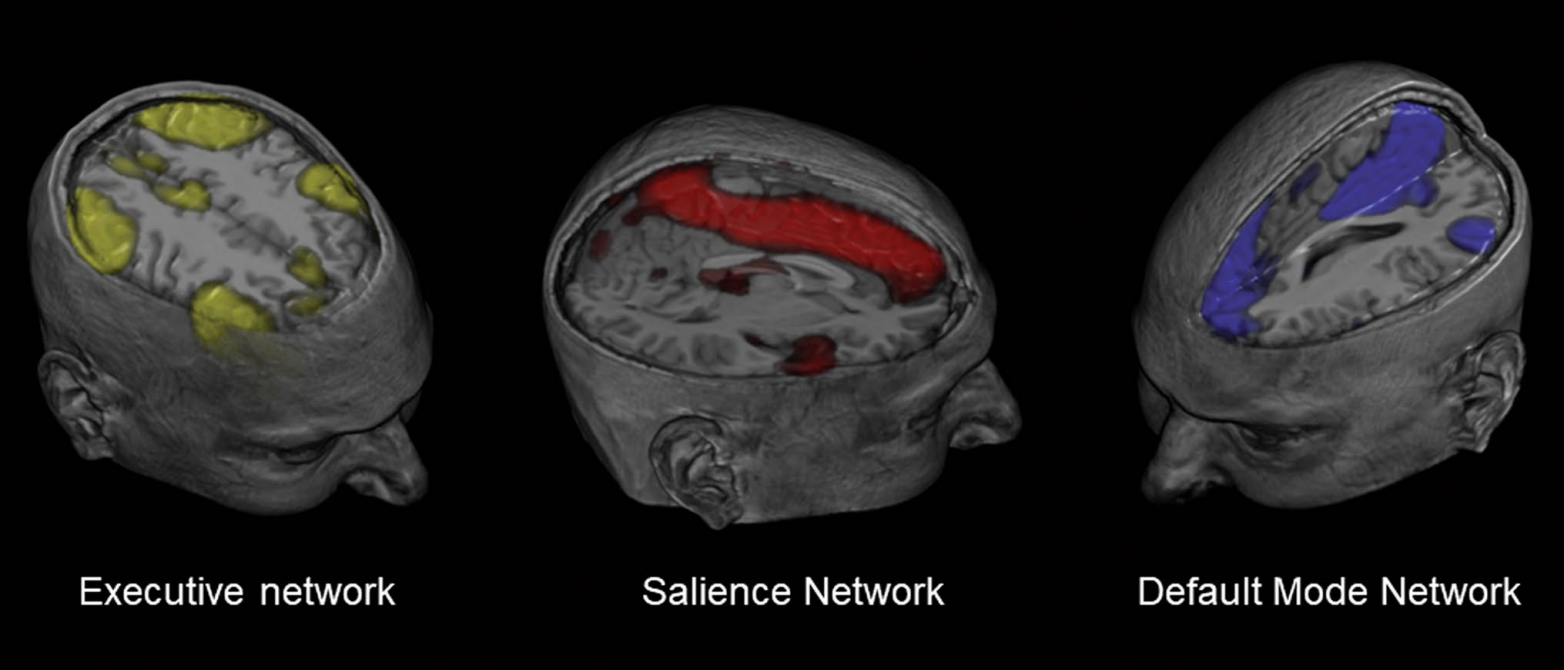
Spatial maps representing neural networks that are important for emotion regulation. The executive network is depicted in yellow (key areas: ventro- and dorsolateral prefrontal cortex, supplementary motor area, and posterior parietal cortex), the salience network in red (key areas: insula and anterior cingulate cortex) and the default mode network in blue (key areas: medial prefrontal cortex, posterior cingulate cortex, and precuneus). Maps are derived from fMRI-data of our own department

Adequate emotion regulation skills are a prerequisite to deal with stressful conditions, such as a mTBI. Although many fMRI-studies have been conducted in non-TBI study samples to investigate neural networks during tasks that challenge certain emotion regulation skills, or during tasks that contain stressful conditions, not much is known about functioning of neural networks during emotion regulation after mTBI [10, 17, 56]. There are two major types of emotion regulation: *top–down* (explicit) and *bottom–up* (implicit) emotion regulation [57]. One of the most important top–down emotion regulation strategies is cognitive reappraisal, which refers to the process of rethinking negative thoughts and changing them into more neutral or perhaps even positive thoughts [58]. Top–down emotion regulation is mainly conducted using the executive network, a network that is also important for cognitive performance [17, 57, 59]. Neural networks that play a pivotal role in emotion regulation are depicted in Fig. 1; notably, the prefrontal cortex occupies a central position in these networks. The default mode network is generally considered a task-negative network, as areas of this network (e.g., PCC, precuneus and medial prefrontal cortex) are consistently deactivated during externally directed mental tasks [60]. Accumulating evidence suggests that these areas are also important for top–down emotion regulation, especially when emotion regulation is stimulated via social support from family-members, friends, or psychotherapists [17, 61]. However, over-activity in this network is often associated with psychopathology such as major depressive disorder [62]. Recently, a study on healthy adults suggested that there is an association between pro-inflammatory cytokines and functional connectivity of the default mode network, which highlights the interaction between stress, inflammation and network dysfunction in psychopathology [63].

In contrast to top–down emotion regulation, bottom–up regulation refers to the implicit processing of salient information that generates emotions, for example a stressful event or stimulus, without engagement of higher order cognitive mechanisms [57, 64]. An example of a bottom–up mental process is when attention is focused to the present internal sensations (e.g., pain, emotions) without cognitively analyzing these sensations. An important network for bottom–up emotion regulation is the salience network, which consists of the insulae and anterior cingulate cortex. The mechanism by which the salience network contributes to effective emotion regulation during stressful conditions, is through generation of emotional awareness and subsequent modulation of executive and default mode network activity [57, 65–67]. The salience network forms an intricate and dynamic link between autonomic bodily arousal responses that accompany emotional and stressful states, and cognitive emotion regulation [68]. Furthermore, HPA-axis reactivity is positively linked to connectivity of the salience network, which highlights the role of this network in modulation of stress responses [67].

Mindfulness, which has also shown to be beneficial in mTBI, engages bottom–up as well as top–down emotion regulation mechanisms, depending on the duration of practice [64, 69]. Mindfulness is a form of meditation, which is derived from Buddhism and involves focusing one’s attention to the present moment, maintaining awareness and control of present thoughts, feelings and sensations, and observing them without judgment [70]. Interestingly, fMRI studies have shown that mindfulness increases activity of areas within the salience network [71], reduces default mode network activity [72], and alters the functional collaboration between the salience, executive, and default mode networks [65].

Resting state functional MRI (fMRI) research from several research groups, including our own, has shown the involvement of the aforementioned neural networks in the presence and persistence of post-traumatic symptoms, anxiety and depression after mTBI [73–77]. Global increases in resting-state functional connectivity in symptomatic patients with sports-related mild TBI seem to have a delayed onset, peaking at approximately 1 week post-injury [78]. Our research group has found that higher resting-state connectivity within the default mode network in het sub-acute phase post-injury, is a possible biomarker for the development of persistent symptoms [74]. However, little is known about neural networks during the actual process of emotion regulation, which may be difficult to perform in daily life for a subgroup of patients with mTBI. This can be tested using emotion regulation fMRI tasks, which are comprised of conditions that evoke either positive or negative emotions. Participants are instructed to apply certain emotion-regulation techniques such as enhancing or suppressing these emotions during these conditions. To our best knowledge, only two studies have been published that used emotion regulation fMRI paradigms in mTBI. One study demonstrated lower activity in the orbitofrontal cortex and superior parietal lobe during bottom–up emotion induction (i.e., condition in which subjects had to judge whether an angry, fearful or neutral face belonged to a male or female), possibly indicating impaired emotion regulation [59]. These findings were related to higher post-traumatic stress scores. The other study showed higher activity of the insula, peri-central gyri, parietal and temporal cortex in emotion enhancement (i.e., condition in which subjects had to try to amplify their negative feelings towards a negatively valenced picture), which may indicate increased vigilance or bottom–up emotional processing, or poor recruitment of areas for emotion regulation [79]. It is still unclear how the executive, default mode, and salience networks function during emotion regulation after mTBI.

## The heart as a biomarker of emotion regulation and stress

So far, it can be concluded that the complex interplay between neural networks that are important for emotion regulation (i.e., executive, default mode, and salience network), the autonomic nervous system, and the HPA-axis, determines an individual’s capacity to deal with stressful situations [56, 80, 81]. The neurovisceral integration model (NIM) states that resting-state heart rate variability (HRV), defined as the variability of time between heartbeats, is an indirect marker of the function of these neural networks [80, 82, 83]. The heart is under constant inhibitory control of the parasympathetic nervous system (vagal nerve tone), which dominates the sympathetic nervous system, keeping the heart rhythm in balance [81]. Simplistically stated, high resting-state HRV primarily reflects higher parasympathetic activity, whereas low HRV reflects activity of the sympathetic nervous system. With respect to the NIM, high resting state HRV is considered a marker of high vagal tone and thus of adequate function of the neural networks. Emotion regulation difficulties and psychopathological states, such as anxiety, depression and post-traumatic stress disorder, are related to lower HRV [80, 81, 83–86]. In addition, higher resting state HRV is associated with better working memory performance in healthy people [87].

The last few years, several studies have been published that report changes in HRV after traumatic brain injury, both at the severe as well as at the mild end of the TBI spectrum [51, 88–93]. Among other things, it has been shown that lower HRV is related to anxiety and depression after TBI [92]. However, most of these studies were sub-optimally designed, and to our knowledge, no study so far focused on HRV in the acute phase after injury, or on the relationship between HRV and function of neural networks (as measured with fMRI).

## The psychophysiological linkage in mTBI

It is still unknown to what extent the various acute biochemical effects are responsible for persistent symptoms and poor outcome after mTBI, and if there is a correlation with psychological factors. More research is required to understand this complex pathophysiological process.

Previous research has shown that traumatic injury to neural, glial or vascular tissue causes the release of brain-specific proteins, and that measurement of levels of these proteins in the acute phase after injury offers some diagnostic value [18–20, 24]. However, not much is known about the role of these proteins in the development of persistent symptoms after mTBI. Another consequence of cellular injury is neuroinflammation, which has barely been investigated in clinical mTBI samples. Neuroinflammation may also develop secondary to high cortisol levels associated with stress. Inter-individual differences in cortisol release acutely after injury can be either linked to the degree of injury to neural structures that regulate the HPA-axis, or to pre-existent hyper- or hypo-reactivity of the HPA-axis [53, 54]. The latter could be associated with pre-existent psychological factors such as the ability to cope with stress or the experience of stressful situations or psychotrauma in childhood. Thus, the extent of the acute stress response probably depends on both physiological as well as psychological factors, although this has never been thoroughly investigated in patients with mTBI.

The interaction between cellular injury, inflammation and stress, mediated by pre-existent coping style or personality, might well be a key mechanism in the persistence of post-traumatic symptoms. Although psychological mechanisms most likely play a dominant role in the development of persistent symptoms, it could be hypothesized that acute physiological effects related to the injury lead to dysfunction of neural networks that are important for emotion regulation. Patients with network dysfunction after mTBI may be prone to developing chronic stress, which facilitates the persistence of symptoms. It is also possible that patients with specific psychological characteristics (for example high neuroticism) have a stronger stress and pro-inflammatory response in the acute phase post-injury. These complex topics can be approached using a combination of personality and coping questionnaires, serum and salivary protein, cortisol and cytokine analyses, and emotion regulation fMRI-paradigms (e.g., cognitive reappraisal and mindfulness tasks). In addition to administering questionnaires, pre-injury stress levels can be assessed using hair cortisol analyses. A prolonged pro-inflammatory state, perhaps due to chronic elevations in cortisol, could contribute to persistent network dysfunction. This can be measured using longitudinal serum or salivary cytokine samples. It has to be realized that causality is a difficult issue (which is inherent in mTBI research), as neural network dysfunction itself may also lead to inflammation. Nonetheless, an intriguing question is to what extent the variance associated with network dysfunction and prolonged inflammation is explained by the injury itself, or by (pre-existent) psychological factors. This directly links to another fascinating question: can mTBI and subsequent stress and inflammatory responses alter coping skills, vulnerability to stress and personality?

Heart rate variability is reflective of autonomic nervous system reactivity related to acute stress, and is also a derivative of the function of neural networks that are responsible for emotion regulation and the ability to deal with stress [83]. Therefore, HRV can be used alongside fMRI-experiments to measure emotion and stress regulation in the acute or later stages after mTBI. For example, a key question is whether acute changes in HRV, measured during presentation at the emergency department, are related to dysfunction of neural networks at a later stage after mTBI.

**Fig. 2:**
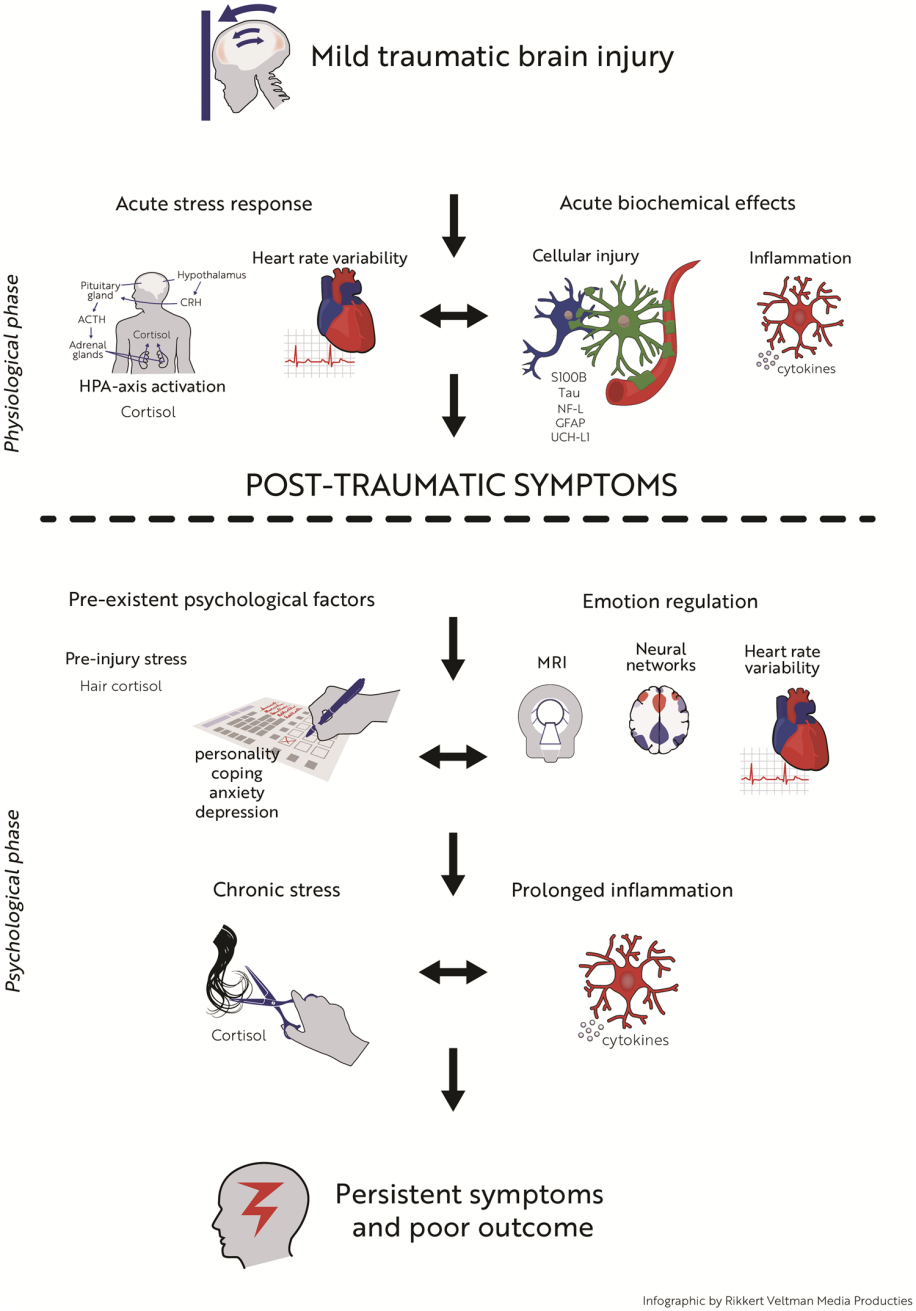
Acute symptoms are probably the result of trauma-induced physiological changes, such as cell injury, inflammation and acute stress. It is thought that maladaptive pre-existent psychological factors (e.g., neuroticism, passive coping, and pre-injury mental distress) impede the ability to cope with acute symptoms leading to the persistence of symptoms. In this infographic we propose a scientific framework that can be used to further study this subject. Infographic was made by Rikkert Veltman Media Producties

In Fig. 2 we present a possible framework that can be used to investigate the interaction between aforementioned physiological and psychological factors. For the sake of clarity, a subdivision was made between the acute ‘physiological phase’ and the more sub-acute/chronic ‘psychological phase’, although we realize that there is much overlap between factors that fall under these phases. The key feature of this infographic is (subjective) post-traumatic symptoms, which develop in the acute phase, and the ultimate goal is to unravel the mechanisms that lead to persistent symptoms and poor outcome.

## Conclusion

In summary, studies so far have suggested that sequelae after mTBI have a complex multifactorial etiology. A better understanding of the interaction between factors is warranted, especially regarding the interaction between acute (neuro)physiological and psychological factors. This understanding is needed to develop tailored psychological or pharmacological interventions in patients with mTBI, which are important future research goals. In our opinion, it is worthwhile to study the influence of acute physiological effects (i.e., cellular damage, inflammation, acute stress) after mTBI on neural networks that are important for emotion regulation, and to examine the possible interactions between these physiological effects and psychological factors such as personality characteristics, coping style, childhood psychotrauma or pre-injury mental distress.
